# Effectiveness of attachment-based family therapy compared to treatment as usual for depressed adolescents in community mental health clinics

**DOI:** 10.1186/s13034-021-00361-x

**Published:** 2021-02-12

**Authors:** Luxsiya Waraan, Erling W. Rognli, Nikolai Olavi Czajkowski, Marianne Aalberg, Lars Mehlum

**Affiliations:** 1 Division of Mental Health Services, Akershus University Hospital, P.O. 1000, 1478 Lørenskog, Norway; 2 Department of Psychology, University of Oslo, Oslo, Norway; 3 Department of Child and Adolescent Mental Health Services, Akershus University Hospital, Lørenskog, Norway; 4 PROMENTA Research Center, Department of Psychology, University of Oslo, Oslo, Norway; 5 Department of Mental Disorders, Norwegian Institute of Public Health, Oslo, Norway; 6 National Centre for Suicide Research and Prevention, Institute of Clinical Medicine, University of Oslo, Oslo, Norway

**Keywords:** Depression, Adolescents, Attachment based family therapy, Efficacy trial

## Abstract

**Background:**

Major Depressive Disorder (MDD) is a disabling mood disorder, profoundly affecting a large number of adolescent’s quality of life. To date, no obvious treatment of choice for MDD in adolescents is available and progress in the treatment of depressed adolescents will have important public health implications. Attachment-Based Family Therapy (ABFT), as the only empirically supported family therapy model designed to treat adolescent depression, aims to repair interpersonal ruptures and rebuild an emotionally protective parent–child relationship.

**Objective:**

To study the effectiveness of ABFT compared with treatment as usual (TAU) delivered within child- and adolescent mental health services (CAMHS) to adolescents with MDD.

**Method:**

Sixty adolescents (86.7% girls), aged 13–18 years (M = 14.9, SD = 1.35), with MDD referred to two CAMHS were randomized to 16 weeks of ABFT or TAU. ABFT consisted of weekly therapy sessions (family/individual or both) according to the treatment manual. TAU was not monitored. Primary outcomes were assessed by blinded evaluators at baseline and post-treatment with the Hamilton Depression Scale (HAMD). Self-reported (Beck Depression Inventory-II, BDI-II) depressive symptoms were assessed at baseline, and after 4, 6, 8, 10,12, 14, and 16 weeks. Analyses were performed according to intent-to-treat principles.

**Results:**

At post-treatment, clinician-rated remission rates on the HAMD (5% in ABFT and 3.33% in TAU, *p* = 1, OR = 1.54, Fisher’s exact test) and self-reported symptoms of depression on the BDI-II did not differ significantly between groups (*X*^*2*^[2, *N* = 60] = 0.06, *p* = 0.97). In both treatment groups participants reported significantly reduced depressive symptoms, but the majority (63.3%) of adolescents were still in the clinical range after 16 weeks of treatment.

**Conclusion:**

ABFT was not superior to TAU. Remission and response rates were low in both groups, suggesting none of the treatments were effective in treating MDD in adolescents. Findings must be viewed in the context of the study’s small sample size, missing data, and implementation challenges. Continued efforts to improve treatment for MDD in outpatient clinics are warranted. Future research should examine moderators of and mechanisms for individual differences to treatment response, as well as the feasibility and cost-effectiveness of implementing treatment models which may require extensive training and expertise to yield clinically meaningful improvements in non-research settings.

*Trial registration* Clinicaltrials.gov identifier: NCT01830088 https://clinicaltrials.gov/ct2/show/NCT01830088?term=Villab%C3%B8&draw=2&rank=1 Date of registration: April 12, 2013

## Background

Depressive disorders entail persistent emotional, biological, and psychological symptoms, accompanied by impaired social functioning [1] and are among the most common psychiatric disorders in adolescents. Nearly 6% of all adolescents meet criteria for Major Depressive Disorder (MDD) at any given time [2]. The prevalence of MDD increases with children transitioning into adolescence [3] and the disorder affects nearly twice as many girls as boys [4]. Experiencing MDD during adolescence increases the risk of further episodes of depression as an adult [5]. MDD is associated with significant disability, morbidity and mortality globally [6], and has been identified as a major risk factor for suicidal behavior and death by suicide [7]. Given the high prevalence and substantial burden of depression in adolescents, developing effective interventions that are feasible to implement in community mental health settings is a high priority.

Variations of cognitive behavioral therapy (CBT) have been most extensively researched, identified as empirically supported psychotherapy approaches [8], and recommended as the first-line treatment for adolescents with moderate to severe depression [9]. With the accumulation of empirical data from randomized controlled trials (RCT) over the past decades, several meta-analyses on treatment of adolescents with depression indicate that Interpersonal therapy—Adolescents (IPT—A) with a considerably smaller evidence base, is a well-established treatment option, along with CBT [8, 10–18]. A recent meta-analysis reported a small effect size (0.29) for CBT and IPT for adolescent depression when compared to active control groups [19] further, a substantial proportion of adolescents fail to remit [19, 20]. Even when treatment is successful, relapse rates are high [21–23]. A network meta-analysis examining the effects of psychotherapies, pharmacotherapies, and their combination in the treatment of moderate to severe depressive disorder in children and adolescents, found Fluoxetine alone or in combination with CBT to be the best choice for the acute treatment [24]. A combination of psychotherapy and antidepressant medication could be the optimal treatment for moderate to severe depression [25, 26]. However, there is little evidence about the benefits and risks of various approaches to treating adolescent depression. The advantages of choosing antidepressant medication over psychotherapy may be limited [27, 28]; the benefit of antidepressant over psychotherapy or a combination approach does not appear to be maintained over time according to a Cochrane systematic review by Cox and collegues [29]. The American Psychological Association (APA) recommendation of Evidence-Based Practice in Psychology (EBPP) [30], defined as the integration of the best available research with clinical expertise in the context of patient characteristics, culture, and preferences, underscores the need for continued efforts to develop and improve treatments.

One consideration in the effort to improve treatment for MDD in adolescents is a greater focus on parental involvement in therapy. Parents can play an important role in the development and maintenance of depressive symptoms in adolescents [31, 32]. Adverse parenting approaches, the level of depressive symptoms in a parent, parent–child conflicts and family dysfunction in general are among the factors that have been associated with adolescents’ depressive symptoms [33]. Family-based interventions, therefore, could have a significant potential to address known risk factors for adolescent depression and could be an effective way of engaging adolescents and their parents in treatment. Attachment-based family therapy (ABFT), developed by Diamond et al. (2002), was designed to help families identify and resolve core family conflicts and attachment ruptures that have inhibited adolescents from trusting their parents and using them as a source of emotional support. ABFT is primarily a process oriented, emotion focused treatment, guided by a semi-structured treatment protocol. The model aims to both improve adolescents’ and parents’ functioning and interactional processes which are important to create a favourable context for individual development.

The empirical support for ABFT is emerging [34–38] and ABFT has been designated as a probably efficacious treatment for adolescents with suicidal ideation based on a study from 2010 [36, 39]. Nevertheless, the evidence is still limited and inconclusive. In the first RCT, where 12 weeks of ABFT was compared to a 6-week waitlist in a sample of 32 adolescents with MDD [35], participants who received ABFT reported significantly lower levels of depressive symptoms and had to a larger extent remitted from depression at post- treatment compared to participants in the waitlist group. Several weaknesses of this study, such as the small sample size, using waitlist as comparison condition, and the duration of the waitlist being only half of the duration of the active treatment, precluded firm conclusions about effectiveness of the treatment. In a second study [36], 66 adolescents were randomized to ABFT or enhanced usual care, similar to TAU in the present study. Participants who received ABFT exhibited greater and faster reduction in suicidal ideation, but ABFT was no more effective in reducing symptoms of depression compared to enhanced usual care at the end of treatment. In a more recent study comparing the effectiveness of ABFT to a family-enhanced nondirective supportive therapy (FE-NST), both treatments produced substantial reductions in depressive symptoms, but ABFT did not outperform FE-NST in reducing suicidal ideation, which was the primary outcome in the study [34]. Furthermore, there was a substantial number of adolescents in both treatment groups who did not achieve remission.

Given the moderate performance of other empirically supported treatments, the continued testing of promising alternative interventions seems warranted. So far ABFT has not been empirically validated in a RCT when implemented outside the context of the treatment developers or in other countries than the USA. In the current study, we aimed to examine the effectiveness of ABFT compared to TAU, an active control treatment, in reducing depressive symptoms in adolescents with MDD, in outpatient clinics, using regular clinical therapists trained in ABFT. Our primary hypothesis was that more adolescents treated with ABFT would remit from depression, compared to adolescents who received TAU.

## Method

### Trial design

The present study was a two-arm, parallel groups randomized trial comparing ABFT with TAU. The study was approved by the regional committee for medical research ethics, South-Eastern Norway and the protocol was pre-registered (http://www.clinicaltrials.gov NCT01830088). Inclusion criteria were (1) a current major depressive episode as defined by the DSM-IV [40], (2) a score above 15 on the Grid Hamilton Depression Rating scale (GRID-HAMD, 40) and (3) currently living with their primary caregiver. Exclusion criteria were a diagnosis of any psychotic disorder, eating disorder, bipolar disorder, intellectual disability or pervasive developmental disorder.

### Participants and procedures

**Fig. 1:**
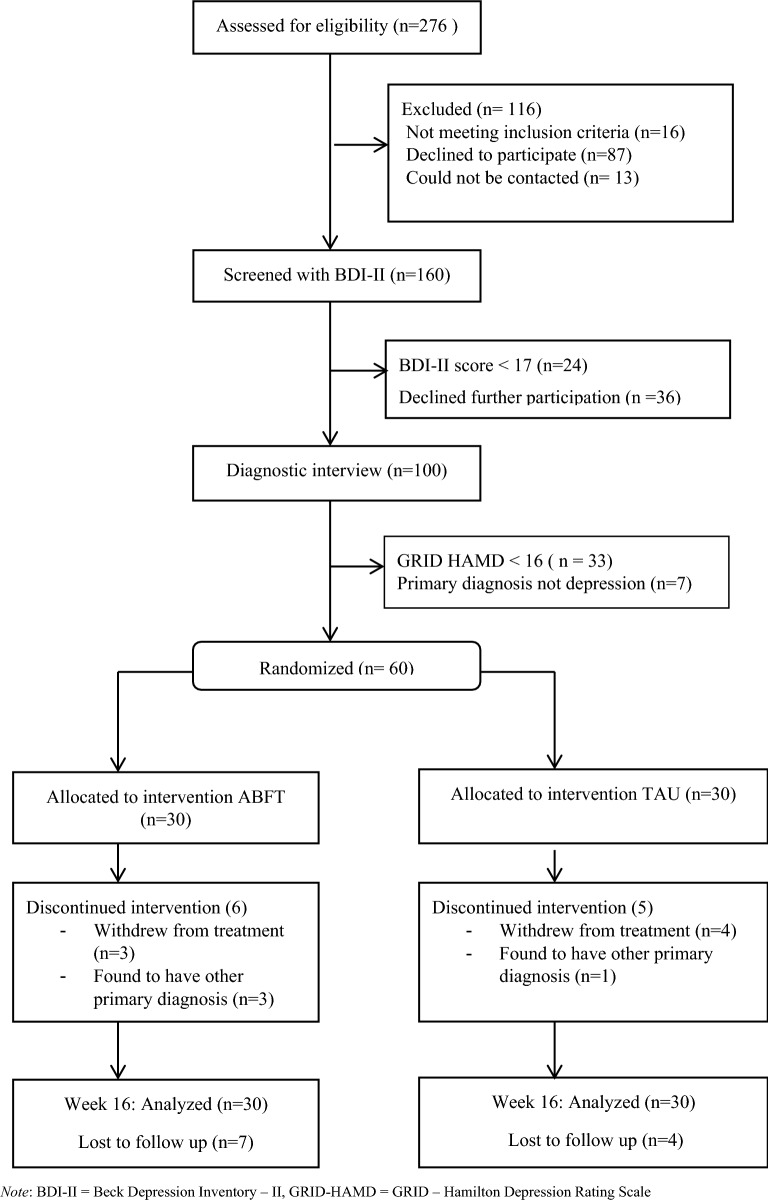
Consolidated Standards of Reporting Trials (CONSORT) flowchart of participants comparing Attachment Based Family Therapy (ABFT) with Treatment as Usual (TAU)

Participating adolescents and their families were recruited among adolescents referred to two child- and adolescent mental health service (CAMHS) clinics in South-Eastern Norway. The clinics were publicly funded, and all treatments were provided free of charge by the universal health insurance system of Norway. During the pre-specified recruitment period, September 2013 to January 2016, all referrals of adolescents (13—18 years) were examined for mentions of depression or core depressive symptoms (depressed mood, anhedonia, or fatigue). Through the use of the Affective Problems subscale on the Youth Self-Report [41] routinely administered by the CAMHS, adolescents with a score > 6 were identified in addition to adolescents who were identified as depressed by their referral letters [42]. Based on these procedures 276 patients were identified, contacted and checked for study eligibility. Altogether 160 adolescents were screened with the the Beck Depression Inventory-Second Edition (BDI-II) after which 100 adolescents and their parents went through a full clinical assessment (see CONSORT diagram, Fig. 1). They met with a study-affiliated clinical psychologist (either the first or second author) at the CAMHS and written informed parental consent and adolescent assent were obtained. Adolescents and parents were then interviewed separately with a semi-structured diagnostic interview, the Schedule for Affective Disorders and Schizophrenia for School-Age Children Present and Lifetime Version (K-SADS-PL) [43] generating DSM-IV-TR diagnoses. All interviews were conducted by the study-affiliated clinical psychologists and were video-recorded. Both parents and adolescents completed self-report measures. If the adolescent met inclusion criteria, the assessing clinician conducted randomization by opening a sealed, numbered envelope containing the treatment allocation. Randomization was stratified by clinic, age (13–15 years and 16–18 years), gender, and severity of depression (GRID-HAMD score of ≤ 24 and ≥ 25). Sixty-one participants were finally randomized to either ABFT or TAU in a 1:1 ratio. Shortly after randomization, one patient withdrew consent resulting in a randomized study sample of 60 participants, 30 in each treatment condition.

Parents and adolescents were given feedback on diagnosis and treatment allocation at the end of the assessment session. The assessing clinician answered questions from parents or the adolescent concerning the assessment, and implemented standard safety procedures to the extent deemed necessary. CAMHS staff were then informed of treatment allocation and given a report of the assessment findings, and assigned the case to a study therapist.

### Treatments

Treatment consisted of 16 weeks of either ABFT or TAU. ABFT consisted of weekly therapy sessions delivered according to the treatment manual by therapists trained in ABFT for the purpose of the trial. TAU was not manualized and the therapists were free to provide the treatment they considered most appropriate. Both treatments were provided for a minimum of 16 weeks, but could be extended depending on the therapists’ assessment of their patient’s needs. For both treatment conditions, results from baseline assessments of psychiatric diagnoses and symptoms were made available to the attending therapist before the first therapy session. If a patient’s data indicated high risk of self-harm or suicide, the study staff immediately notified the patient’s therapist. Two participants were on antidepressant medication during the trial and they were on a stable dose for at least 12 weeks before being enrolled in the study.

### Therapist characteristics and training

Over a period of 2 years, 25 (88% female) therapists delivered the treatments; 19 clinical psychologists, 2 medical doctors, 2 clinical pedagogues, 1 clinical social worker and 1 psychiatric nurse. Therapists delivered either ABFT or TAU only. Therapists varied in their theoretical orientation, including eclectic (40%), cognitive (16%), psychodynamic (4%), or family-oriented (4%) therapy. The therapists had an average of 7.2 years of clinical experience working with adolescents (*SD* = 5.73, range 0–18). Eight therapists were trained in ABFT for the purpose of the study. Training consisted of a day-long introductory seminar, followed by a 3-day workshop, as well as reading the treatment manual. Therapists were required to have completed one case of ABFT under supervision before treating study patients. All ABFT sessions were videotaped for supervision purposes. Therapists had ongoing supervision by an experienced ABFT therapist, reviewing video recordings of therapy sessions. Supervision of therapists was planned as weekly sessions. The original PI on this study was not certified as an ABFT therapist or trainer, but had very solid training in the approach. He served as the main clinical supervisor. Many of the weekly supervision sessions had to be conducted as peer supervision, when the supervisor was occupied or otherwise unavailable. Sometimes, supervision was conducted as a combination of peer supervision and discussion with the main supervisor by phone. To increase objectivity, the developers of ABFT provided some training, but had no involvement in the supervision and minimal involvement in the project.

TAU therapists were recruited from the regular staff of the CAMHS, and treated patients in the trial as part of their regular caseload. TAU was non-monitored and access to supervision varied by clinical experience, but all therapists could discuss cases in multidisciplinary teams.

### Assessments and measures

For the duration of the treatment, patients completed self-report measures electronically every other week using a secure online platform (CheckWare Assessment Systems) [44]. Some self-report measures were administered occasionally as paper and pencil questionnaires by the treating clinicians, because of technical difficulties. Post-treatment assessment at 16 weeks was conducted by independent raters (clinical psychologists trained for this purpose) blinded to treatment allocation. Both the main diagnosis and comorbid psychiatric diagnoses were determined based on the K-SADS at baseline, combining information from the adolescent and parent interviews. Interrater reliability was determined by blind scoring of 28 randomly selected videotaped interviews. Interrater reliability for depressive diagnoses based on the K-SADS interview in this study was κ = 0.56. The primary outcome measure was severity of depressive symptoms measured by the clinician-rated GRID-HAMD and participants’ self-report on the BDI-II. BDI-II, a widely used 21-item self-report inventory, was used to assess the severity of depressive symptoms every other week throughout the duration of the trial. Internal reliability was α = 0.94. GRID-HAMD was measured at pre- and post-treatment. GRID-HAMD has been shown to have good psychometric properties as a measure of depression severity [45, 46]. The average Intraclass correlation coefficient (ICC) for GRID-HAMD scores in this study was 0.89, based on a two-way mixed consistency. GRID-HAMD scores are classified as no depression (0‐7); mild depression (8‐16); moderate depression (17‐23); and severe depression (> 24) [47]. Clinical response is defined as improvement in GRID-HAMD total score by ≥ 50% from baseline and remission from depression as GRID-HAMD score < 5. Suicidal ideation was measured with the Suicidal Ideation Questionnaire-Junior (SIQ-Jr) [48], and was used in this study in the multiple imputation process.

### Changes to the protocol

A power analysis was conducted before the trial start, based on previous ABFT studies. Assuming a 10% attrition rate from the acute phase, an intra-subject correlation of 0.5 on the longitudinal measures, an adjusted alpha of 0.001 to accommodate for multiple comparisons, and 80% power, a sample size of N = 100 would allow us to detect a small effect size (d = 0.27). Our final sample, however, consisted of 60 adolescents and parents. As a consequenze of small sample size and missing data, the use of more stringent alpha levels in subsequent analyses were abondend, and multiple group comparisons were not conducted. Further, only the most important variables were included in subsequent analyses. According to the protocol we planned to assess the primary and secondary outcomes at weeks 12, 24 and 48. We originally intended to adopt a four week waiting period from randomization to treatment start but this turned out not to be feasible due to the severity of the depressive symptoms for many patients. The treatment period was extended from 12 to 16 weeks, and the first outcome assessment was conducted at week 16 and not 12 as specified in the protocol.

### Statistical analysis

GRID-HAMD scores at 16 weeks post randomization were missing for 22 of 60 participants (36.7%). In some cases, adolescents actively declined to provide data. In other cases, when participants did not turn up for scheduled assessment appointments they were not targeted for renewed appointments to collect their data for practical reasons, such as lack of interviewer capacity. In both cases, we considered it likely that the probability of having missing outcome data was conditional on patient characteristics, and hence non-ignorable [49]. We used baseline data to analyse correlates of missingness calculating polychoric correlations between a binary coding of missingness for week 16 GRID-HAMD, and the sumscores of an extended set of baseline variables available in the dataset [50]. We found missingness to be positively correlated with negative self-statements, insomnia and suicidal ideation and negatively correlated with self-reported motivation for talking to a therapist. As we found several theoretically plausible predictors of missingness, we made the assumption that missingness was sufficiently conditional on observed variables, and conducted multiple imputation to handle the missing data [49],using the package ‘mice’ version 3.3.0 for R version 3.5.1, with RStudio IDE [51–53]. Multiple imputation yields several complete datasets with variation in imputed values across the datasets preserving the uncertainty due to data being unobserved. Analyses are repeated across all the imputed datasets and results from these multiple analyses are then pooled for interpretation, allowing estimates of the influence of missing data on the obtained parameter estimates (for a highly accessible treatment of multiple imputation, see [54]). Conducting multiple imputation of variables composed of multi-item scales can be challenging. Ideally, imputations should be conducted at the level of individual items [55]. However, with several multi-item scales, the number of predictors in the imputation model will often surpass what is feasible with a limited sample size, as the number of predictors approach the number of observations. All variables in the model to be estimated using the multiply imputed data need to be included as predictors in the imputation, and including other variables as auxiliary predictors increases the plausibility of the necessary assumption of missingness being conditional on variables in the imputation model [52]. Following recommendations of Eekhout and colleagues [56], we set up our imputation with separate imputation of the individual items of the GRID-HAMD only, and passive imputation of the total score, recalculating it iteratively each time the component scores were imputed.

We used baseline GRID-HAMD score, treatment condition and BDI-II and Suicidal SIQ-Jr scores at 16 weeks as predictors in the imputation model for GRID-HAMD scores on theoretical grounds [49]. We examined both individual items and scale scores from other measures, including measures completed by the parents, to select auxiliary predictors for each GRID-HAMD item, as well as for the BDI-II and SIQ-Jr scores at 16 weeks [54]. Predictors for imputing any missing values in these predictor variables themselves were selected using the ‘quickpred’ function of the mice package [52]. We used the ‘midastouch’ version of predictive mean matching as the imputation method, which has better performance in small sample contexts [57]. We imputed 40 datasets, using 50 iterations of the algorithm. Convergence of the imputation algorithm was assessed by visual inspection of traceplots and found to be satisfactory [49].

**Fig. 2:**
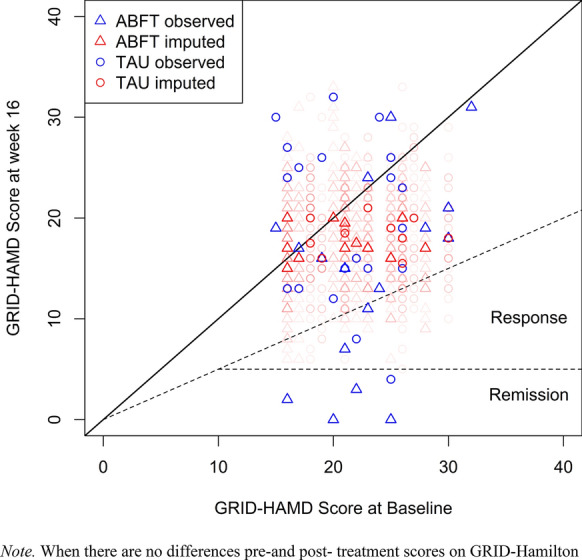
Modified Brinley Plot, a scatter plot to visualise individual change on clinician rated depressive symptoms from before and after therapy. When there are no differences pre-and post- treatment scores on GRID-Hamilton Depression Rating Scale (GRID-HAMD), data points are aligned on the 45° line. Points above this line represent depression scores that are higher at week 16 than at baseline (and reversely for points below the 45° line). The shaded symbols represent the uncertainty of the imputed scores. The dashed lines represents clinical cut off points for remission and response

Data were analysed by intent-to-treat (ITT) principles. The primary hypothesis was tested with Fisher’s Exact Test. Linear mixed models were fitted to test whether treatment condition was significantly related to change in scores over time on the primary and secondary outcome variables. For primary outcome measured by GRID-HAMD, which had only two timepoints for the observations, we fitted the model to all the imputed datasets and then pooled the resulting estimates. Pooled estimates according to Rubins rules are reported [49]. BDI-II had 55% missing across eight timepoints, primary outcomes with multiple observations were analyzed in a mixed modeling framework, handling missing observations using maximum likelihood estimation [50]. Variable selection for multilevel analyses was implemented to minimize the information criteria (IC). Since a group mean can conceal changes at an individual level, a Brinley plot [58] was used to visualize within-subjects effects, from pre- to post-treatment score (Fig. 2). The Brinley plot is based on the multiply imputed data set, and the uncertainty of the imputed scores is visualized in the plot.

Analyses were conducted using R (version 3.5.1) and the package lme4 (version 1.1–17) [51, 59] and an alpha level of 0.05.

## Results

Table 1 summarizes the baseline sociodemographic data and clinical characteristics of the participants by treatment condition. Mean age was 14.9 (SD = 1.35) and 52 (86.7%) of the participants were female. For the sixty adolescents who were included, 43 fathers and 57 mothers participated, all adolescents had at least one parent participating in the study. Upon study entry 50% had one or more comorbid psychiatric diagnosis in addition to MDD. In both treatment groups all patients completed the 16 weeks of treatment. However, three participants (5%) in the ABFT and four (7%) in the TAU condition withdrew from the study, that is, they declined to provide outcome data at the end of treatment.

**Table 1:** Baseline demographic and diagnostic data in adolescents (N = 60) allocated to attachment based family therapy or treatment as usual

Variable		Treatment condition	
		ABFT (n = 30)	TAU (n = 30)
Age, years (SE)		15.03 (1.35)	14.77 (1.36)
Gender, % (n)	Female	90 (27)	83.3 (25)
Dropout, % (n)	Excluded	7 (2)	3.3 (1)
	Drop out	10 (3)	13.3 (4)
Ethnicity, % (n)	Norwegian	100 (30)	96.7 (30)
	Skandinavian other than Norwegian	0 (0)	3.3 (1)
Living with, % (n)	Both parents	29.6 (8)	36.7 (11)
	Two home family	18.5 (5)	13.3 (4)
	Father (and partner)	18.5 (5)	13.3 (4)
	Mother (and partner)	33.3 (9)	33.3 (10)
	Other	0 (0)	3.3 (1)
Psychiatric comorbidity, % (n)	Dysthmia	3.3 (1)	0 (0)
	Any anxiety disorders^a^	43.3 (13)	46.7 (14)
	Obsessive–Compulsive Disorder	6.7 (2)	6.7 (2)
	Externalizing disorder	0 (0)	13.4 (4)
	PTSD	3.3 (1)	3.3 (1)
	Eneuresis	3.3 (1)	6.7 (2)
	No comorbidity	53.3 (16)	46.7 (14)
Depressive symptoms, mean (SD)	BDI-II	34.23 (7.34)	36.21 (9.84)
	GRID- HAMD	21.87 (4.61)	21.92 (4.07)

*BDI-II* Beck Depression Inventory II, *GRID-HAMD* GRID-Hamilton Depression Rating Scale

^a^Includes social phobia, specific phobia, agora phobia, generalized anxiety disorder, anxiety disorder NOS, obsessive compulsive disorder

^b^Includes oppositional defiant disorder, attention deficit/hyperactivity disorder

### Primary outcomes

There was no significant difference in the remission rate between ABFT and TAU participants over the 16-week treatment period. Only five (8.33%) adolescents remitted; 3 (5%) in ABFT and 2 (3%) in TAU (*p* = 1, OR = 1.54, Fisher’s exact test). To examine the association between clinician-rated depressive symptoms at posttreatment and treatment condition, a series of linear mixed model analyses with maximum likelihood test were performed (Table 2). Time was entered as fixed effect in model 1, along with a random effect for each adolescent. Time had a significant effect on depressive symptoms *t*(90.29) = -3.87*, p* < 0.001. Model 1 fitted significantly better than a null model (*p* < 0.001). In Model 2, time and treatment condition were entered as fixed effects, along with an interaction effect of time and treatment condition. Model 2 did not fit significantly better than model 1 (*p* < 0.98). Mean depression scores were reduced from 21.8 (SE = 1.14) at baseline to 17.36 (SE = 1.6) at week 16, but only the coefficient for time (*p* < 0.01) had a significant impact on depressive symptoms. The interaction term of time and treatment group was not significant, *t*(92.115) = 0.17, *p* = 0.86. There was no significant fixed effect of treatment group (ABFT/TAU), *t*(112.04) = 0.042, *p* = 0.97. Adjusting for age and sex did not change any of the models.

**Table 2:** Linear Mixed model of depressive symptoms measured by GRID-HAMD at week 16

Fixed effect	Null Model				Model 1					Model 2			
	β	CI	*t*	*p*	Β		CI	*t*	*p*	β	CI	*t*	*p*
Intercept	19.71	(18.41, 21.01)	30.05	–	21.83		(20.26, 23.41)	27.40	–	21.80	(19.55, 24.05)	19.20	0.00
Time					− 4.25		(− 6.43, − 2.07)	90.29	–	− 4.44	(− 7.62, − 1.25)	− 2.77	0.01*
ABFT										0.07	− -3.11, 3.25)	0.04	0.97
ABFT:Time										0.38	(− 3.96, 4.71)	0.17	0.86
Model comparison – Wald													
Model 1 vs. Null model						0.0001*							
Model 2 vs. 3						0.98							

*ABFT* Attachment Based Family therapy, *GRID-HAMD* GRID-Hamilton Depression Rating Scale

Mean clinician-rated depressive symptoms pretreatment was 21.87 (*SD* = 4.61) in ABFT and 21.92 (*SD* = 4.07) in TAU, mean post-treatment scores were 17.81 (*SD* = 1.34) in ABFT and 17.36 (*SD* = 1.45) in TAU.

**Fig. 3:**
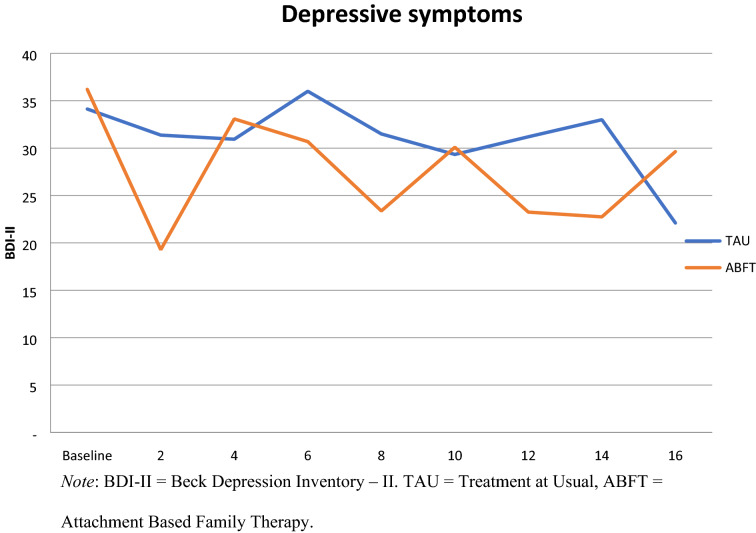
Self- reported depressive symptoms by treatment at baseline and after 2, 4, 6, 8, 10, 12, 14,16 weeks of of treatment

Self-reported depressive symptoms were analyzed through a set of mixed models. First, time was fitted as linear, squared or exponential fixed effect. The linear effect of time was the best fit for the data. Then fixed effect of treatment allocation and a treatment by time interaction term was added. There were no significant differences in rates of change in self-reported symptoms of depression between ABFT and TAU (Table 3). The mean bi-weekly reduction in BDI-II score was—0.94 (SE = 0.42) over the 16 weeks of treatment. The effect of treatment allocation and the interaction between time and treatment allocation were not significant. Given the high missingness in these data, there is an uncertainty about whether the erratic nature of the self-reported depressive symptoms shown in Fig. 3. truly is a valid clinical observation or a result of data collection problems. An independent samples t-test was performed to test if the number of sessions attended by the adolescent differed between ABFT and TAU during the 16 weeks. Adolescents in the ABFT treatment group (*M* = 28.66, *SD* = 8.32) received significantly more sessions than adolescents in the TAU condition (*M* = 19.73, *SD* = 6.49, *t*[47.19] = 4.31 *p* = 0.001).

**Table 3:** Linear Mixed Model of self-reported depressive symptoms measured at baseline and after 2, 4, 6, 8, 10, 12, 14,16 weeks of the treatment

Fixed effect	Null Model		Model 1		Model 2	
	β	CI	β	CI	Β	CI
Intercept	32.07	(29.41, 34.75)	34.64	(32.46, 36.81)	34.67	(31.58, 37.77)
Time			– 1.017	(– 1.62, – 0.42)	– 0.94	(– 1.81, – 0.09)
ABFT					– 0.0.07	(– 4.41, 4.28)
ABFT:time					– 0.144	(– 1.35, 1.05)
Random effects						
σ^2^_Intercept_	83.98		41.35		41.35	
σ^2^_Time_			1.85		1.85	
Residual	7.71		6.4		6.4	
Model inf						
AIC	1608.08		1561.40		1565.34	
BIC	1618.22		1581.68		1592.38	
Log-Likelihood	– 801.04		– 774.70		– 774.67	
χ2 (df)			52.67 (3)		0.06 (2)	
Pr(> Chisq)			< 0.001 ***		0.97	

Self reported depressive symptoms measured by Beck Depression Inventory-II

*ABFT* Attachment Based Family therapy

### Clinical significance

Figure 2, the Brinley plot illustrates how individual adolescents scored pre- and post-treatment on clinician rated depressive symptoms. Only 16.6% of the adolescents were rated as in the nonclinical range (GRID-HAMD < 15) at post-treatment, 63.3% adolescents remained relatively unchanged in the clinical range, and 16,6% adolescents were rated as having more severe symptoms at the end of treatment.

## Discussion

The findings of the present study indicated that ABFT was no more effective than TAU in the treatment of adolescents with depression. Both clinician-ratings and self-reports showed reductions in depressive symptoms from pre- to post-treatment, but there were no differences in the outcomes of the two treatment conditions. Only five out of 60 youth showed full clinical remission after 16 weeks of treatment, three in ABFT and two in TAU.

The lack of differences between ABFT and TAU is consistent with findings by Diamond and colleges [34, 36], who reported no differences between ABFT and enhanced usual care or non-directive supportive treatment in terms for remission rate, clinical response or reduction of depressive symptoms at the end of treatment. However, in their studies, the adolescents’ reduction in depressive symptoms was clinically significant. In the present study, very few adolescents achieved full remission at the end of treatment, and importantly, most of the participants showed only small improvements in their level of depressive symptoms and were still considered clinically depressed based on independent clinical ratings. Evaluations of other treatment approaches for adolescent depression suggest that remission and response rates vary greatly. There is limited evidence about the comparative effects of different treatment approaches [29]. Most meta-analyses point to a superior effect of medication in combination with psychotherapy [24, 25]. Nonetheless, one would expect a larger number of adolescents in remission after a full course of effective psychotherapy [11, 12, 61, 62], than what was observed in our study. The lack of improvement for adolescents receiving TAU observed in the present study is not entirely surprising. A majority (60%) of adolescents seen by specialists in CAMHS clinics and receive non- monitored TAU make little or no measurable improvement on any indicator of individual level-change [63]. These findings suggest that depression in adolescents is hard to treat in a CAMHS setting, where clinicians get limited training in effective treatments and there is a continued need for improving treatment effectiveness [64]. On the other hand, there is some evidence to suggest that contact with mental health services reduces the future likelihood of depression compared to those without contact with mental health services in adolescents [65].

There may be several possible explanations for the observed low treatment response in the present study. Treatment duration of 12 to 16 weeks is a common dosage of treatment in treatment evaluations. Studies evaluating CBT, IPT or a combination of CBT and Fluoxetine, with the same treatment duration have found these treatment approaches to be more effective than TAU and other active control conditions for adolescents with depression [11, 25, 27, 61], suggesting that it is reasonable to expect some clinical improvement following 16 weeks of treatment. In the present study, adolescents in the TAU group received considerably fewer treatment sessions than adolescents in the ABFT group, within the same time frame. A higher number of therapy sessions per week have been found to be associated with better treatment outcome in one previous study [66]. Even if this finding is debated, some would argue one could expect to observe a greater indication of improvement after 16 weeks of ABFT than what we found given the dose difference between the treatment conditions. One possible explanation for the low treatment response may be that implementation challenges precluded the therapists from learning and implementing the model in a good enough manner.

Another obvious possible interpretation of our findings may be that ABFT is simply not a more effective treatment for adolescent depression compared to TAU. The attachment-based components of psychotherapy embedded in ABFT did not result in a superior psychotherapy [64]. A likely interpretation of the failure of finding any statistically or clinically significant change in the investigated treatments is that none of these treatments are effective in reducing depressive symptoms. Our sample was comparable to samples in other trials in terms of severity of depressive symptoms, comorbidity and other important factors [11, 34, 36]. It seems unlikely that the pretreatment level of depressive symptoms can explain the lack of improvement at the end of treatment in participants of our trial. Earlier evaluations of ABFT have relied on samples consisting of adolescents from ethnic minorities, many living in one-parent households, or households with income below the poverty line [34, 36]. These factors differenciate previously studied populations from the present study in several important ways. Adolescents from low income families may experience greater relational conflicts [67, 68] and may therefore benefit more from a treatment approach targeting family relations and conflicts specifically[69].

Depressive disorders are heterogeneous, and the underlying mechanisms of depression may vary greatly among adolescents. A manualized therapy, focusing on relational bonds may not be suitable for everyone. ABFT targets conflicts between adolescents and their parents specifically, as well as attachment ruptures, and works well on resolving issues around such problems [38]. ABFT may thus be more suitable for adolescents whose depressive disorder is related to problems in the parent-adolescent relationship, and who experience conflicts and high level of stress in their families. However, empirical efforts to identify factors which may guide treatment selection have not yet provided any conclusive evidence [70]. Given the large number of adolescents with depression who do not respond sufficiently to a first-line treatment, it is necessary with continued efforts to identify factors that may moderate the treatment effects. Demonstrating significant differences between active psychotherapy approaches in efficacy and effectiveness studies is difficult and often requires a large sample size. Head to head comparison of treatments often results in no differences between treatment groups even in fully powered well executed trials [12, 16, 71, 72]. Our results must be viewed in the context of the study limitations and strengths. First, our sample size was relatively small. The study, with its final sample size, was not adequately powered to detect small differences in effect size between the two active treatments. Prior to data collection a power analysis was conducted, but the target sample size was not achieved. Although our study was underpowered, which could lead to the erroneous conclusion that the intervention groups do not differ, the final sample size would still enable us to identify a clinically meaningful and statistically significant difference between the two treatment groups. On the other hand, the convergence of both outcome measures and consistency with observations from other studies strengthen the findings. Implementation challenges and challenges in collecting data lead to high level of missingness despite that there was a low rate of premature termination of treatment. Even though we used recommended methods for handling missing data and followed ITT principles, we cannot completely rule out that there is a degree of selective attrition which may have impacted our results. Third, ABFT was a new intervention to the clinics, and ABFT may thus be more susceptible to barriers to implementation. ABFT was implemented relatively shortly before the trial onset, while TAU had the advantage of being a well-established practice at the clinics. Training of clinicians to adequately perform ABFT may need more time, supervision and practice than what was offered. Changes to the supervision structure may have affected the clinicians negatively. Fidelity was not assessed, although the issue was addressed in supervision with the ABFT therapists. Without proper adherence measurement it is difficult to determine whether the method was used adequetly by the therapists in the trial. The trial supervisor discussed implementation of the protocol with the developers, and reviewed recorded and live therapy sessions using the ABFT adherence measure. TAU was not monitored. Despite several limitations of the present study, we emphasize the importance of reporting the results of this trial. This study has high external validity as it was conducted in general mental health sevices, with a clinically referred population and few exluction criteria. Publication bias and over-representation of positive trials introduces bias into meta-analyses because critical data are not available to the field, which consequently misinforms researchers, clinicians and policymakers [73]. Importantly, a failure to report less than ideal results can lead to ineffective treatments being implemented, negative findings needs to be transparent to give a clear picture of the research field.

## Conclusion

Findings suggest that in this study ABFT was not more effective than TAU.” Few adolescents achieved full remission and many reported symptoms of depression in the clinical range at the end of treatment. Data collection and implementation of the treatment model was challenging. There is currently insufficient evidence to reach any firm conclusions reagarding the role of ABFT in psychological therapy for depressed adolescents. It is important in future research to examine possible moderators of treatment outcome and mechanisms of change in order to better tailor treatment to the individual. The cost-effectiveness and feasibility of implementing a treatment model such as ABFT, which may require extensive training and expertise in order to yield clinically meaningful improvement, should also be examined.

## Data Availability

The data that support the findings of this study are available from Akershus University Hospital, but restrictions apply to the availability of these data, which were used under license for the current study, and so are not publicly available. Data are, however, available from the authors upon reasonable request and with permission of Akershus University Hospital and the Regional Committee for Medical Research Ethics, South-East Norway.

## Abbreviations

MDD: Major Depressive Disorder
CBT: Cognitive Behavioural Therapy
IPT-A: Interpersonal therapy for Adolescents
RCT: Randomiced Controll Trial
ABFT: Attachment Based Family Therapy
FE-NST: Family-Enhanced Nondirective Supportive Therapy
TAU: Treatment as Usual
CAMH: Child and Adolescent Mental Health Service
GRID-HAMD: GRID – Hamilton Depression Rating Scale
BDI-II: Beck Depression Inventory – II,
K-SADS-PL: Scheduale for Affective Disorders and Schizophrenia for School-Age Children, Present and Lifetime Version
SIQ-JR: Suicidal Ideation Questionnaire-Junior
ITT: Intent- to- treat
IC: Information crietria
